# Emotional control in selected somatic and psychiatric diseases

**DOI:** 10.1186/s12888-023-05321-7

**Published:** 2023-11-03

**Authors:** Agata Orzechowska, Paulina Maruszewska, Małgorzata Gałecka, Philip Hyland, Daniel Boduszek, Piotr Gałecki, Katarzyna Bliźniewska-Kowalska

**Affiliations:** 1 Department of Adult Psychiatry, Medical University of Lodz, Lodz, 91-229 Poland; 2 Department of Psychotherapy, Medical University of Lodz, Lodz, 91-229 Poland; 3 Department of Psychology, Maynooth University, Maynooth, Co. Kildare Ireland; 4 Faculty of Psychology, SWPS University, Wrocław, 53-238 Poland; 5 Department of Psychology, University of Huddersfield, Huddersfield, HD1 3DH UK

**Keywords:** Emotional control, Emotion regulation, Somatic Disease, Mental Illness

## Abstract

**The aim:**

was to assess the level of subjective control of emotional states among patients treated for dermatological and gastrointestinal somatic diseases compared to those with depressive and anxiety disorders. The results were related to the analyzed dimensions of emotion regulation in healthy subjects.

**Materials and methods:**

The reports of the conducted studies were compiled for a total of 310 people, including 120 patients diagnosed with a somatic disease (psoriasis, rosacea, irritable bowel syndrome, and gastroesophageal reflux), as well as 96 patients diagnosed with depressive disorders and 30 patients with anxiety disorders. The control group consisted of healthy subjects (64 individuals). To assess the psychological variables analyzed, the subjects completed the Emotion Regulation Questionnaire developed by J. Brzeziński.

**Results:**

The study showed that the patients suffering from a chronic somatic symptom disorder, similarly to those treated for depression and anxiety disorders, differed from the healthy individuals in most aspects of emotional control. The patients with dermatological and gastrointestinal diseases differed statistically significantly from the patients with depression and the patients with anxiety disorders in relation to three dimensions of emotional control. Patients with a somatic disease are characterized by higher emotional and rational motivation, lower emotional resilience and lower emotional arousal.

**Conclusions:**

A chronic disease co-occurs with the emotional sphere of a person’s daily functioning. Regardless of the diagnosis in terms of somatic disorders and mental illnesses, the way in which emotional states are controlled can be an important factor in the onset of the disease, coping with it as well as the treatment process.

## Introduction

Each person is characterized by a tendency to react emotionally in a certain way, which is typical and fairly independent of the situation. This tendency involves three components of emotions, i.e., the individual’s subjective feelings, physiological changes, and the expression of emotion as presented in the person’s behavior. The ability to control behavior subject to emotion is inherent in these components. Emotional control is the process by which people can influence what emotions they experience, when the emotions are exacerbated, and how they evaluate and expresses them. Emotional control is an indicator of emotional maturity and the ability to constructively express one’s emotional states depending on the situational context [1, 2].

Emotional and affect control is a component of emotion regulation, which is a broader term—although it is sometimes used interchangeably with emotional control, and includes the processes responsible for initiating, modeling, and maintaining the experience as well as expression of emotions. Emotion regulation refers to the processes of self-control and to correcting the ways in which emotions are expressed, changing their content and intensity, modulating emotion-related behaviors, and serving to undertake adaptive and goal-oriented action strategies. Emotional control is responsible for strengthening, weakening or changing an emotional response. A person with properly developed emotional control is able to inhibit their emotions in some situations and freely reveal them in others [3–6].

Although emotional responses are usually appropriate to the demands of the environment and allow for an effective action, there are times when a person’s experienced emotions lead to behavior that is contrary to their needs and goals. Features of dysfunctional experience and regulation of emotions are usually observed and analyzed in people suffering from mental illnesses, such as affective disorders, anxiety disorders or personality disorders, fitting into the clinical picture of these disorders. It has been shown that such people reveal problems in understanding emotions and their control and, as a result, may have periodic or long-term difficulties in forming close emotional ties, or their ties are characterized by ambivalence, conflicts or overdependence on others [4, 7]. Of the most common and costly brain diseases listed in terms of treatment, more than 60% of the social and economic costs are generated by mental disorders from the group of depressive and anxiety disorders, being one of the most common reasons for seeking help from specialists [8]. The two disorders share similar biological mechanisms that determine their underlying causes, as well as common psychological mechanisms, including emotional and cognitive phenomena such as chronic worry and ruminations, fixed negative thought patterns about oneself, one’s surroundings and the future, resulting in interpersonal problems [9].

Recently, emotion regulation processes have also been considered in the context of chronic somatic diseases, not only as a reflection of the negative consequences of the disease, such as its inconvenience and chronic nature, but also as a risk factor involved in their formation. The emotional sphere of the personality, including mainly tension and negative emotions and difficulties in regulating them properly, in conjunction with difficult and stressful situations, co-occur with a decrease in the body’s immunity [10, 11]. The psychophysiological mechanism of emotion may be an important and common risk factor relevant to the course of both mental and somatic chronic diseases. The prevalence of unpleasant emotions and the inability to discharge negative emotional tension increase vulnerability to stress and adversely affect health. Thus, the control of emotions in difficult situations, which undoubtedly include the struggle with a chronic disease, is important for the process of coping with the course of the disease [12, 13].

The regulation of emotions takes place through three brain regions working together. The brainstem structures are responsible for the most elementary, innate and unconscious drive reactions (arousal or inhibition and autonomic responses). The limbic system, including mainly the amygdala and hippocampus, modifies emotional responses depending on incoming external stimuli—the environment. The prefrontal cortex, on the other hand, is responsible for feelings (conscious emotions) and control over emotions [14, 15]. The formed biological structures of the human brain are subject to various external impacts, which can act supportively or negatively at each developmental stage determining our resilience and coping ability [16]. Thus, fixed abnormal behavioral traits can lead to dysregulation of the functioning of specific brain structures and a constant pro-inflammatory response by the immune system, triggering a cascade of reciprocal feedback. These changes can consequently lead to mood disorders, including depression, personality disorders, psychoses, anxiety disorders and dementia syndromes [7].

Psychoneuroimmunology is the study of the interactions among behavioral, neural, and endocrine, and immune processes. The brain communicates with the immune system through autonomic nervous system and neuroendocrine activity. Both pathways generate signals that are perceived by the immune system via receptors on the surface of lymphocytes and other immune cells. An activated immune system, in turn, generates cytokines that are perceived by the nervous system. Thus, bidirectional connection between the brain and the immune system reinforces the hypothesis that immune changes could mediate some of the effects of psychological factors on health and disease [17].

Immunological disorders and chronic inflammation, which can consequently cause similar abnormal psychological reactions and disorders as in mental illnesses, can also occur in the course of some chronic somatic symptom disorders [13, 18].

The aim of the study was to analyze emotional control variables in several somatic diseases (dermatological- psoriasis, rosacea- and gastroenterological—irritable bowel syndrome, gastroesophageal reflux disease) compared to patients with selected mental illnesses (depression and anxiety disorders), against healthy subjects.

## Materials and methods

Surveys among patients with somatic disease and mental illness were conducted in dermatology and gastroenterology departments, as well as in the psychiatric ward of university hospitals in Lodz, Poland. The somatic disease group included patients with psoriasis (30 patients), rosacea (30 patients), irritable bowel syndrome (IBS) (30 patients), and gastroesophageal reflux disease (GERD) (30 patients). The mean age of all individuals surveyed in this group was M = 38.91 years, standard deviation (SD) = 11.74, minimum age = 18 years, maximum = 55 years.

The patients with depressive disorders, including diagnoses of depressive episode (F 32 according to ICD-10 criteria) and recurrent depressive disorder (F 33) − 96 patients, and patients with anxiety disorders (30 patients) in the form of phobias (F 40) and other anxiety disorders (F 41), were under hospital treatment. The mean age of all the people surveyed in this group was M = 44.59 years, standard deviation (SD) = 11.97, minimum age = 18 years, maximum = 61 years.

Patients with mental comorbidity, somatic disease other than the disease entities selected for the study, including neurological, inflammatory and oncological, were excluded from the study. The authors did not interfere with the diagnosis and treatment process at any stage of the study. The research was performed individually by the authors using the research methods described below. Medical data on the course of the disease were obtained directly from the patients and from attending physicians (with the patients’ consent).

The comparison group (64 people) included those who did not meet the criteria for a diagnosis of mental disorders and somatic diseases. The mean age of all the people surveyed in this group was M = 32.77 years, SD = 10.56, minimum age = 20 years, maximum age = 61 years.

Detailed sociodemographic data describing all the subjects invited to the study, including patients with somatic disease and mental illness, and the control group of healthy subjects, are provided in Tables 1 and 2.

**Table 1:** Characteristics of the study groups part 1- mental problems versus healthy control

Variable	Depression (N = 96)	Anxiety disorders (N = 30)	Healthy subjects (N = 64)
**Gender**	66 F 30 M	17 F 13 M	38 F 26 M
**Age**	47.07 ± 11.12 (min. 18 / max. 61)	36.63 ± 11.25 (min. 19 / max. 60)	32.77 ± 10.56 (min. 20 / max. 61)
***Age depression versus anxiety disorders***	*Z = 4.12* *p = 0.000 (p < 0.05)* Cohen’s d = 2.15	***Age anxiety disorder versus healthy subjects***	*Z = 1.71* *p = 0.088*
*Age depression versus healthy subjects*	*Z = 6.68* *p = 0.000 (p < 0.05)* Cohen’s d = 3.15		

F—female, M—male; N- number; ±—standard deviation; Z—Mann-Whitney U test; p—statistical significance

**Table 2:** Characteristics of the study groups part 2 – somatic diseases

Variable	Psoriasis (N = 30)	Rosacea (N = 30)	Gastroesophageal reflux (GERD) (N = 30)	Irritable bowel syndrome (IBS) (N = 30)
**Gender**	13 F 17 M	18 F 12 M	12 F 18 M	22 F 8 M
**Age**	38.33 ± 12.81 (min. 18 / max. 55)	43.43 ± 9.92 (min. 27 / max. 55)	41.43 ± 10.96 (min. 19 / max. 55)	32.43 ± 10.57 (min. 19 / max. 55)
**Total**: ***Age depression versus somatic diseases***	*Z = 5.12* *p = 0.000 (p < 0.05)* Cohen’s d = 3.34	***Age anxiety disorders versus somatic diseases***	*Z = 0.95* *p = 0.344*	
*Age somatic diseases versus healthy subjects*	*Z = 3.39* *p = 0.001* Cohen’s d = 2.89	*Age mental illnesses versus somatic diseases*	*Z = 3.82* *p = 0.0001* Cohen’s d = 3.43	

*F—female, M – male; N- number; ±—standard deviation; Z—Mann-Whitney U test; p—statistical significance*

The selection of participants for the study group was random. Each subject gave written informed consent to participate in the study. Each study was conducted in accordance with the rules of the Data Protection Act, and its design was approved by the Bioethics Committee of the Medical University of Lodz under the following applications: RNN/882/11/KB and RNN/383/11/KB.

Emotion Regulation Questionnaire developer by J. Brzeziński [19] was used for the study. The method contains 45 statements rated on a four-point scale. It is used to measure subjective control of emotions in difficult situations, which consists of five dimensions:

- *expressivity control* (KE)—measures an individual’s ability to control the outward manifestations of experienced emotions (body movements, gesticulation, facial expressions). A high score indicates excessive expressivity control, a low score shows underdeveloped expressivity control.
- *emotional and rational motivation* (MER) *-* indicates the type of motivation of the individual, i.e., the type of control of one’s own behavior, which on the one hand can be emotional (impulsive) and on the other rational (controlled and thoughtful). A high score indicates controlled and thoughtful behavior, and a low score shows impulsive behavior.
- *emotional resilience* (OE)—measures the ability of the subject to prevent behavioral disorganization under the influence of experienced positive and negative emotions, as well as the ability to influence the source of emotions, allowing to suppress the current emotional process. A high score indicates resilience to emotions and excessive control over one’s own behavior; a low score signifies non-resilience to emotions and an inability to control the behavior associated with them.
- *situation control* (KS)—indicates the individual’s ability to control emotogenic (emotion-triggering) situations, to perceive and interpret them appropriately, taking into account whether the individual easily enters or avoids emotion-triggering situations. A high score indicates excessive control of the situation manifested mainly in the form of anxiety, and resulting from defensively oriented perception and interpretation. A low score means that the individual enters into various emotion-triggering situations too easily, without thinking about the consequences of doing so.
- *emotional arousal* (PE)—measures the threshold of general emotional arousal and illustrates the degree to which it is easy to enter an emotional state after being exposed to emotion-triggering stimuli. The lower the score on this scale, the more difficult it is for the individual to enter an emotional state, i.e., the less emotionally excitable they are [19].

The subjects provided other sociodemographic variables, like gender, age, duration of disease, by filling out a short questionnaire prepared by the study authors.

STATISTICA 13.3 PL software was used for statistical analysis of the results obtained. A two-sided critical area was assumed during the statistical verification of the hypotheses. In order to choose the type of measurement, an analysis of the variables under study was conducted, which showed that the hypothesis of conformity to normal distribution should be rejected. In order to demonstrate the statistical significance of the association of the analyzed variables among the patients treated for somatic, affective and anxiety disorders and among the healthy individuals, a statistical analysis was performed based on non-parametric tests, including the Mann-Whitney U test and the Spearman’s rank correlation coefficient. The significance level of p < 0.05 was adopted in all statistical methods used.

## Results

**Table 3:** Duration of disease

	Number of patients (somatic disease)	Number of patients (mental illness)
**over 10 years**	36	18
**6–10 years**	19	12
**3–5 years**	31	38
**1–2 years**	25	27
**up to a year**	9	31
**TOTAL**	120	126

The study groups of patients with somatic symptom disorders were dominated by those with chronic disease—more than 10 years and 3 to 5 years. Among the patients with mental illness, those affected for 3 to 5 years and patients with illness for 1 to 2 years were the largest group (Table 3).

**Table 4:** Emotional control: somatic diseases versus mental illness

	Mean (M) somatic disease	Mean (M) mental illness	Z	p	Cohen’s d
**KE**	13.97	14.74	1.53	0.126	
**MER**	16.68	13.50	6.40	0.000 *(p < 0.05)*	3.42
**OE**	13.57	14.71	2.52	0.012	3.43
**KS**	17.65	17.66	0.12	0.908	
**PE**	14.00	18.36	7.55	0.000 *(p < 0.05)*	3.42

Z—Mann-Whitney U test; p—statistical significance; Cohen’s d- effect size measure; KE—expressivity control; MER—emotional and rational motivation; OE—emotional resilience; KS—situation control; PE—emotional arousal

The patients with dermatological and gastrointestinal diseases differed statistically significantly from the patients with depression and the patients with anxiety disorders in relation to three dimensions of emotional control. The people with somatic disease are characterized by higher emotional and rational motivation (MER), lower emotional resilience (OE) and lower emotional arousal (PE) (Table 4).

**Table 5:** Emotional control: somatic diseases versus healthy people

	Mean (M) somatic disease	Mean (M) healthy subjects	Z	p	Cohen’s d
**KE**	13.97	13.52	0.67	0.503	
**MER**	16.68	15.00	2.89	0.004	2.89
**OE**	13.57	14.55	1.57	0.117	
**KS**	17.65	14.78	5.71	0.000 *(p < 0.05)*	2.88
**PE**	14.00	12.45	2.73	0.006	2.89

Z—Mann-Whitney U test; p—statistical significance; Cohen’s d- effect size measure

KE—expressivity control; MER—emotional and rational motivation; OE—emotional resilience; KS—situation control; PE—emotional arousal

Table 5 shows that—compared to the healthy individuals—the patients treated for dermatological and gastrointestinal diseases have statistically significantly higher levels of emotional and rational motivation (MER), situation control (KS) and emotional arousal (PE).

**Table 6:** Emotional control: mental illness vs. healthy subjects

	Mean (M) mental illness	Mean (M) healthy subjects	Z	p	Cohen’s d
**KE**	14.74	13.52	2.13	0.033	2.82
**MER**	13.50	15.00	2.55	0.011	2.82
**OE**	14.71	14.55	0.51	0.613	
**KS**	17.66	14.78	6.42	0.000 *(p < 0,05)*	2.82
**PE**	18.36	12.45	7.62	0.000 *(p < 0,05)*	2.81

Z—Mann-Whitney U test; p—statistical significance; Cohen’s d- effect size measure

KE—expressivity control; MER—emotional and rational motivation; OE—emotional resilience; KS—situation control; PE—emotional arousal

The patients with mental disorders—compared to healthy subjects—recorded the highest number of statistically significant differences in the analyzed dimensions of emotional control. They have higher levels of expressivity control (KE), situation control (KS) and emotional arousal (PE) than the healthy subjects. The dimension of emotional and rational motivation (MER) scored significantly lower compared to the healthy people. The results described are shown in Table 6.

**Fig. 1:**
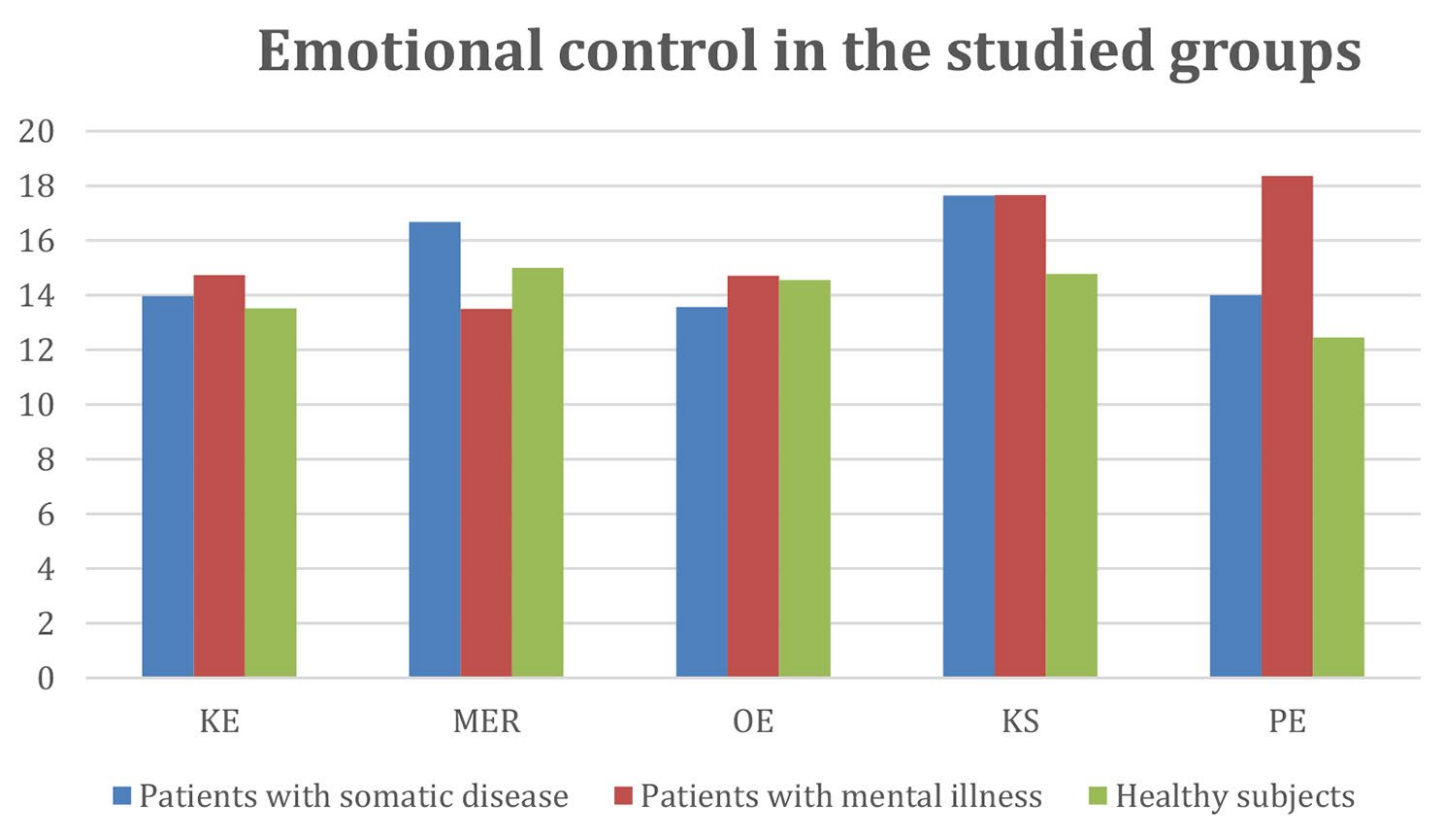
Emotional control scores among all study subjects. KE—expressivity control; MER—emotional and rational motivation; OE—emotional resilience; KS—situation control; PE—emotional arousal

**Fig. 2:**
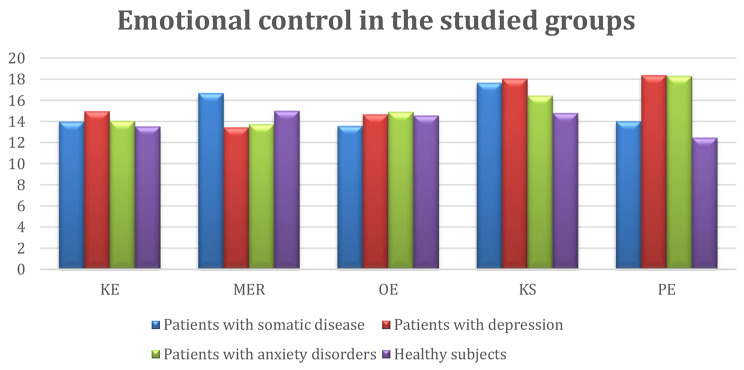
Emotional control scores among all study subjects. KE—expressivity control; MER—emotional and rational motivation; OE—emotional resilience; KS—situation control; PE—emotional arousal

A graphical presentation of the obtained results of the differences between the average values in each group is shown in Figs. 1 and 2.

All subgroups of somatic symptom disorders and mental illnesses were compared with the healthy subjects. Tables and figures include selected results of the statistical analysis without breakdown by patient group.

On the situation control (KS) subscale that measures an individual’s ability to control emotion-triggering situations as well as their correct perception and interpretation, most of the subjects with somatic disease and mental illness scored higher than the healthy subjects. This indicates a defensive attitude and a tendency to control the situation in the form of anxiety. In the group of patients with anxiety disorders (M = 16.43; SD = 2.65) and patients with psoriasis (M = 16.20; SD = 3.00), these scores were slightly lower compared to the others. Those with irritable bowel syndrome (M = 18.23; SD = 3.57) and rosacea (M = 18.90; SD = 3.19) recorded similar results to the patients with depression (M = 18.04; SD = 3.04).

The dimension of emotional arousal (PE), which refers to the ease of entering an emotional state under the influence of emotion-triggering stimuli, was dominant in the group of patients with mental illness. On the other hand, expressivity control (KE) (controlling the outward manifestations of experienced emotions) and emotional resilience (OE) (assessing the ease of entering an emotional state under the influence of emotion-triggering stimuli) in terms of the mean scores obtained were most similar in all the patients studied compared to the control subjects.

Interestingly, in the emotional and rational motivation (MER) subscale, higher scores—compared to those with depressive and anxiety disorders, and even compared to the healthy people—were obtained by those with somatic diseases, i.e., psoriasis (M = 17.33; SD = 4.44), rosacea (M = 15.60; SD = 3.29), gastroesophageal reflux (M = 17.30; SD = 3.15), irritable bowel syndrome (M = 16.50; SD = 3.56). A higher score on this scale indicates motivation to engage in more rational and less emotional behavior in emotion-triggering situations.

### Age, duration of Illness and gender of study subjects

Subjects with a diagnosis of chronic somatic symptom disorders were statistically significantly different in **age** from those with mental illness and from those in the control group. After a detailed statistical analysis, the group of patients with anxiety disorders was not statistically significantly different from those with somatic disease (all ‘dermatological’ and ‘gastrointestinal’ patients combined) and from the healthy subjects, as can be seen in Tables 1 and 2, presenting the characteristics of the subjects. This has to do with the nature of the diseases in question—the mean age of onset typical of the disease entity and the availability of the subjects during hospitalization and outpatient visits during the course of the study.

Among those with somatic diseases, the **age** of the subjects co-varied statistically significantly with a small number of the emotional control variables discussed. In the patients with psoriasis, a positive correlation was for emotional and rational motivation (R = 0.38; p = 0.036) and situation control (R = 0.38; p = 0.040). A negative correlation for expressivity control (R= -0.41; p = 0.024) was confirmed among the patients with gastroesophageal reflux. Age did not correlate statistically significantly with the variables analyzed in the group of patients with rosacea and irritable bowel syndrome as well as among patients with anxiety disorders. In contrast, among those with depressive disorders, a significant positive correlation extended only to situation control (R = 0.28; p = 0.005). Age co-occurred statistically significantly with expressivity control (R = 0.29; p = 0.021) and emotional and rational motivation (R = 0.26; p = 0.037) among the healthy subjects.

**Duration of illness** did not correlate statistically significantly in the anxiety disorder, psoriasis and irritable bowel syndrome groups. In the group of patients suffering from depression, a significant negative correlation was for emotional resilience (R= -0.20; p = 0.048). A significant negative correlation was also confirmed for emotional arousal (R= -0.37; p = 0.044) among the patients with rosacea. The patients suffering from gastroesophageal reflux had a significant negative correlation in situation control (R= -0.48; p = 0.007) and emotional arousal (R= -0.37; p = 0.044). In all the cases described, longer duration of illness co-occurred with lower intensity of the listed dimensions of emotional control.

**Gender** differentiated the subjects (patients with somatic disease, mental illness and the control group) in a statistically significant way with reference to only two dimensions concerning emotional control, namely situation control (Z = 2.95; p = 0.003) and emotional arousal (Z = 4.25; p = 0.000), which had higher levels among women. The differences between men and women were greatest among patients with depression. They addressed the following dimensions of emotional control: expressivity control, situation control and emotional arousal, with higher results for women in all the dimensions mentioned.

## Discussion

Measuring emotional control takes on particular importance in the context of scientific reports that prove the existence of a relationship between the way emotions are expressed and the occurrence of mental, somatic and psychosomatic diseases [20].

There are few reports in scientific studies comparing patients with chronic somatic disease to patients with psychiatric disorders in terms of control of emotional states. Most reports in the literature refer to common factors that link somatic diseases to depression or the co-occurrence of psychiatric disorders with chronic physical illness, mainly in a biological context [18, 21, 22].

The association of somatic diseases with depression and anxiety is grounded in the presence of biological factors common to these diseases. Both depression and anxiety disorders, as well as a number of diseases of civilization (hypertension, coronary heart disease, diabetes and even dementia), share a common immunological background. An increase in immune system activity and the adverse metabolic changes described above can be seen in each of the aforementioned diseases [23]. The same brain regions that are particularly sensitive to the consequences of weakened immune defenses are responsible for both emotion dysregulation and cognitive dysfunction, i.e., the anterior and medial cingulate cortex, the dorsolateral and ventromedial prefrontal cortex, the anterior insula, and the amygdala [24, 25]. This would also suggest similar emotional reactions in these diseases. In some situations, the difference between somatic diseases and mental illnesses can blur in terms of emotional reactions.

The reason for the co-occurrence of anxiety disorders with somatic diseases as with depression may be related to common biological and behavioral processes involved in the development of these types of diseases. There are also similarities in the pathophysiology of anxiety and selected somatic diseases or somatization tendencies [26, 27].

The accepted models of disease formation primarily take into account biological factors relating to the genetic background, biological dysregulation conditions involving the activities of individual systems and organs of the body, which the authors of the presented paper did not directly address. It is pointed out that psychological and environmental factors—in addition to biological factors—play a significant role in the development of diseases, which in their theoretical assumptions emphasize the importance of personality traits (the totality of the way of thinking, experienced emotions and behavior), early human experience and adaptation to environmental demands, or the way of cognitive elaboration of one’s own experiences and evaluation of oneself. Certain personality traits can promote illness, while at the same time, changes in selected personality traits can be a consequence of the disease process [28].

Deficits in the regulation of emotional control can include and simultaneously co-occur with the phenomenon of alexithymia, which is described as a disorder of cognitive and affective processes that limits a person’s access to his own mental states and awareness of his own emotions. Alexithymia is treated as a stable personality trait of patients, which, along with other personality factors, predisposes to the occurrence of various somatic and mental illnesses. The limited ability of a person with alexithymia to become aware of and process his own emotions using cognitive processes leads to both a focus on somatic sensations accompanying emotional arousal and a compulsive, poorly controlled response to negative stimulation [29].

In the analysis of the study variables presented in this publication, the patients with skin diseases and the patients with gastrointestinal disorders differed statistically significantly from those with psychiatric disorders and from the healthy subjects on three dimensions of emotional control. People with somatic disease are characterized by higher emotional and rational motivation, lower emotional resilience and lower emotional arousal than those with mental illness. Compared to the healthy individuals, the patients treated for dermatological and gastrointestinal diseases have statistically significantly higher levels of emotional and rational motivation, situation control and emotional arousal. The patients with mental disorders—compared to the healthy subjects—recorded the highest number of statistically significant differences in the analyzed dimensions of emotional control. They have higher levels of expressivity control, situation control and emotional arousal than the healthy subjects. They scored lower on the dimension of emotional and rational motivation compared to the healthy people. The results described for the people with mental illness remain consistent with many other studies addressing this topic. In addition, regardless of statistical significance, there was similarity in the individual factors comprising emotional control on the dimensions that differentiate people with somatic disease and mental illness from healthy individuals.

The literature on this issue indicates that emotion regulation disorders are important in the development and course of many mental illnesses such as depressive disorders, bipolar affective disorder, borderline personality disorder, generalized anxiety disorder, and eating disorders [30]. Numerous studies on emotion regulation—regardless of the very conceptualization of the construct and the methods used—unequivocally point to the differences that exist between people with a diagnosis of a mental disorder and the population of healthy people [31, 32]. Emotion regulation in patients who reveal mental disorders is usually characterized by multiple deficits. Their emotional reactions are usually excessive, violent, marked by long-lasting negative emotional states; they perform an adaptive function insufficiently, and most often are clearly dysfunctional for a given situational context. The literature provides a big number of reports on the association of maladaptive emotion regulation strategies with the severity of psychopathological symptoms [33, 34]. Empirically, the negative impact of emotion suppression on health—expressed as an increase in the level of psychopathological symptoms—has been established. At the same time, emotion acceptance has been proven to reduce the severity of symptoms.

It has been repeatedly shown that emotion suppression is related to the level of depressive symptoms and leads to an increase in negative emotions. Studies evaluating the relationship between the ability to control emotions and the severity of symptoms of depressive and anxiety disorders have shown that the more people control, suppress and devalue their emotions, the higher their levels of anxiety and depression symptoms. Conversely, the more accepting they are of their emotions—both negative and positive—the less severe the symptoms of depression and anxiety [35]. The results in subsequent publications clearly indicate reduced levels of emotion acceptance, increased suppression, more frequent ruminations, and negligible use of positive reformulation strategies in these patients compared to healthy individuals [36–38]. Research also shows that suppression of positive emotions is associated with anhedonia, increased negative emotions and elevated levels of depression [34, 35].

Dermatological and gastroenterological diseases are among those with complex etiologies in which a significant role is played by both biological and psychological factors. In most cases, these diseases cause bothersome symptoms that impede daily functioning and may even result in unsightly changes in appearance, affecting the patient’s body image and self-esteem, and to a large extent determine the patient’s social relations [21, 38–40].

In our own studies from 2008 to 2010 [41, 42] the determinants of emotional control among people treated for gastroesophageal reflux disease and irritable bowel syndrome were assessed in conjunction with levels of experienced stress, coping styles and perceived anxiety. The results of the analysis conducted in 2008 showed that the patients studied did not differ statistically significantly in terms of the psychological factors analyzed, but the variables examined co-occurred with the severity of selected somatic symptoms. The results obtained in 2010, on a slightly larger number of patients, showed the prevalence of unfavorable aspects of emotional control in the group of patients diagnosed with IBS, as well as higher levels of perceived stress and anxiety as an ongoing personality trait and currently experienced condition.

Also in reports from 2009 [43], in which 70 people with dermatological diseases were surveyed, the Coping Inventory for Stressful Situations by R.H. Moss and Emotion Regulation Questionnaire by J. Brzeziński were used to assess psychological factors. The type of skin disease only partially differentiated the patients studied in terms of the psychological factors analyzed. They included control of emotion-triggering situations, emotional arousal and cognitive avoidance. The women surveyed differed from the men in terms of control of emotional expressivity and control of emotion-triggering situations, and in terms of overall emotional resilience. Among all the patients studied, positive aspects of emotional control correlated with less frequent use of less favorable stress coping strategies. The results presented suggest that the unfavorable aspects of emotional control may be related not so much to the type of disease entity, but to the disease itself, which may be close to the research results achieved in the current work.

Atroszko et al. [44] studied 31 patients aged 18–78 suffering from arterial hypertension. They compared the values obtained on the Emotion Regulation Questionnaire by J. Brzeziński, the Type A-Framingham scale and the Emotionality, Activity and Sociability Temperament Questionnaire with a control group of men aged 18–87. The results gave a consistent picture of the emotional functioning of a man with hypertension as a person with a high tendency to react with dissatisfaction and anger, striving to maintain control in emotion-triggering situations to avoid experiencing emotions, especially avoiding failure and feeling tension, and characterized by a tendency to be competitive and hostile. The authors of the study concluded that the tendency to frequently experience negative emotions may be a mediating factor between personality traits and the development of hypertension.

The relationship between emotional functioning and somatic diseases has been a topic of scientific research for several decades. The mechanism of this relationship is explained on the basis of various models that describe the relationship between emotions and physiological responses, mediated by other psychological factors such as stress and personality traits. Individual susceptibility to reacting with negative emotions and emotional control processes is of interest from this perspective [23, 45]. Measuring emotional control takes on particular importance in the context of scientific reports that prove the existence of a relationship between the way emotions are expressed and the occurrence of mental, somatic and psychosomatic diseases. In our study, people with somatic symptom disorders scored higher on the emotional and rational motivation subscale and on most dimensions of emotional control compared to people with depressive and anxiety disorders, and even relative to healthy people. This may be related to the nature and course of chronic diseases. A chronic disease is a stressor of a special nature, requiring specific measures to overcome the symptoms of the disease and cope with chronic difficult emotional states. Coping which involves cognitive and behavioral efforts to transform or eliminate burdens and associated emotional tension may secondarily—in the course of a long-term disease—affect the intensification of the tendency to emotional control observed in the results we obtained [46].

### Emotional control versus age, onset of Disease and gender

In our study, we showed the existence of heterogeneous relationships between age and various dimensions of emotional control across groups. Age differentiated the subjects, but did not co-occur in a statistically significant way with a large number of emotional control variables. In the vast majority of them, selected dimensions of emotional control increased with age.

Other studies addressing this topic also indicate that the ability to control emotions increases with age, as the ability to understand and name one’s own emotions associated with the development of social interactions rises. One study found that factors such as emotional suppression and aggression control, indicating a tendency toward less emotional expression, are highest in the group of people over 55. This phenomenon is explained by the fact that older adults may be better able to understand and accept their inner subjective experiences, condense them, and do not feel such a strong need to express their emotions. In addition, perhaps older people suppress the expression of negative emotions more, or experience fewer negative emotional reactions compared to younger people [47]. The more mature a person is, the more effective they are able to make the use of emotion-focused coping strategies such as distancing, self-control and positive reappraisal [48]. Some researchers conclude that as the subjects get older, they approach a more favorable continuum in terms of the listed psychological factors, which may be related to emotional maturity and a sense of life stability, a formed personality and self-esteem, and acceptance of their own illness [49].

In our own research and considering all the groups studied, we have shown that women enter a state of emotional arousal with greater ease and at the same time show a greater ability to control expressivity. In contrast, in the group of depressive patients, women were characterized by greater emotional arousal, with stronger expressivity control and situation control. In the literature we find data indicating that women score higher on emotional expressivity than men in a group of patients suffering from various types of chronic diseases. The results also support the view that emotions are culturally constructed and context dependent. Cultural changes and social consequences that affect age and gender roles can have a direct impact on the ways in which emotions are experienced and expressed [47].

Duration of disease did not correlate in a statistically significant way in the group of patients with anxiety disorders, psoriasis and irritable bowel syndrome. In the group of patients with depression, rosacea, gastroesophageal reflux, it was shown that longer duration of disease co-occurred with lower intensity of selected dimensions of emotional control. In the case of patients with depression, emotional resilience decreased with longer duration of disease, while in the case of patients with somatic disease, emotional arousal diminished mainly. This may suggest a tendency toward better emotional functioning with the duration of the disease in the case of a somatic condition, but not a mental one, which is understandable given the nature of mental disorders. A study by Kossakowska et al. [50] showed that the duration of a dermatological disease was not correlated with the severity of negative emotional control, such as anger, depressive mood and anxiety, but was strongly correlated with the severity of disease progression as assessed by clinicians [50]. In other studies conducted with patients suffering from psoriasis, it was shown that disease duration was associated with a greater degree of disease acceptance and ability to regulate emotions. With longer duration of disease, the tendency to use denial strategies decreased [51]. In contrast, in a group of people with rheumatoid arthritis, it was shown that with disease duration women behaved more ambiguously—they became emotionally unstable and worried, while at the same time persevering and reconciled to life. A greater number of adaptive behaviors were observed in men [52]. The duration of the disease is indirectly related to the intensity of the disorder’s symptoms.

### Limitations

The heterogeneity of the comparison groups of subjects, in terms of the number of subjects studied and demographic variables, may be considered a limitation of the presented project. They varied significantly in terms of age, which is related to the prevalence of specific disease entities in the age range and the availability of healthy individuals who consented to the study. The study results should be treated with caution. Further research in this area should continue on larger group sizes in specific disease entities.

## Conclusions

A somatic disease and mental disorder of a chronic nature entails a fundamental and usually inauspicious change in a person’s life in the form of persistent ailments that cause suffering and restrictions on the performance of important social roles—both family- and work-related. Both categories of disease entities are closely related to the function of the nervous system and to the human mental sphere. Emotional processes, which have a significant impact on the way of thinking, behavior and physiological reactions, are a factor that determines the overall functioning, as well as the quality of life of an individual.

Comparing patients with somatic disease to patients with mental illness in terms of controlling emotional reactions raises new possibilities for covering the described groups with similar therapeutic interventions that pay attention to the patient’s emotional sphere. The importance of various disease factors should be studied and taken into account in the treatment process, with the aim of selecting the most optimal therapeutic tasks.

Control of emotional reactions, which is the variable analyzed in the current study, is one of the established personality traits, as we described in the discussion of the work. Other well-established personality traits and their contribution to somatic and psychiatric disorders will become the subject of our upcoming studies. In the given study, the authors also did not address the therapeutic side of the described phenomenon, and due to the significant number of patients affected by this problem, it requires much more research attention in the future.

## Data Availability

Upon request from corresponding author.

## List of abbreviations

IBS: irritable bowel syndrome
GERD: gastroesophageal reflux disease
